# Comparative chloroplast genomics of wild-type *Panicum miliaceum* cv. ATL1 and its M_4_ mutant line: insights for molecular breeding applications

**DOI:** 10.1186/s12870-025-06999-5

**Published:** 2025-08-04

**Authors:** Ayushi Kumar, Ravikesavan Rajasekaran, Iyanar Krishnamoorthy, Senthil Alagarswamy, K. Chandrakumar, Sowmya Pulapet, Kesavan Markkandan, Selvaraju Kanagarajan, Manikanda Boopathi Narayanan

**Affiliations:** 1 Centre for Plant Breeding and Genetics, Tamil Nadu Agricultural University, Coimbatore, Tamil Nadu 641003 India; 2 Department of Plant Physiology, Tamil Nadu Agricultural University, Coimbatore, Tamil Nadu 641003 India; 3 Oneomics Private Limited, Bharathidasan University Technology Park, Khajamalai Campus, Tiruchirappalli, Tamil Nadu India; 4 Department of Plant Breeding, Swedish University of Agricultural Sciences, Lomma, Sweden; 5 School of Science and Technology, The Life Science Centre, Örebro University, Örebro, Sweden; 6 Department of Plant Biotechnology, Centre for Plant Molecular Biology and Biotechnology, Tamil Nadu Agricultural University, Coimbatore, Tamil Nadu 641003 India

**Keywords:** Chloroplast genome, *Panicum miliaceum*, Proso millet, Poaceae, Phylogenomic analysis, Codon bias, SSR analysis, Mutagenesis

## Abstract

**Background:**

Proso millet (*Panicum miliaceum L.*), one of the oldest domesticated crop, remains an underexploited resource with significant potential for nutrition and yield. With evolving breeding perspectives, genomic knowledge is increasingly vital for developing new crop varieties. However, the limited genomic information on indigenous proso millet hinders its full utilization. This study addresses this gap by compiling chloroplast genome (cp. genome) data for the native variety ATL1 and its mutant derivative TN*Pm*PEM 001, aiming to facilitate the development of new varieties.

**Results:**

Both *Panicum miliaceum* cv. ATL1 and TN*Pm*PEM 001 chloroplast genomes exhibited the characteristic quadripartite structure. While they shared identical total lengths (139 837 bp), small single-copy (SSC: 12 795 bp), large single-copy (LSC: 84 522 bp), and inverted repeat (IR: 20 560 bp) regions, these metrics diverged from the reference genome, which displayed a total length of 139 826 bp, with distinct SSC (12 574 bp), LSC (81 682 bp), and IR (22 785 bp) regions. While soybean, cotton, sunflower, and pea constituted outgroups, the phylogenetic analysis showed a tight link between ATL1, TN*Pm*PEM 001 and reference cp. genome as well as with little millet. The identification of protein-coding genes regulating photosynthesis components (photosystems I and II, NADH dehydrogenase, cytochrome complexes, rubisco, and ribosomal/tRNA/rRNA genes) in both investigated cp. genomes provides critical insights into the genomic basis of photosynthesis efficiency in underutilized C_4_ crops like proso millet, a key trait for improving stress-resilient sustainable agriculture. Additionally, 11 unique simple sequence repeat (SSR) markers, exclusively identified in the mutant derivative, offer novel tools for marker-assisted breeding programs targeting agronomic trait enhancement.

**Conclusions:**

These findings address critical gaps in proso millet genomics, particularly the limited molecular resources for Indian landraces. The mutant-derived SSRs and structural variants offer actionable targets for enhancing yield stability under variable photoperiods, a priority for climate-resilient proso millet breeding in marginal agroecosystems.

**Supplementary Information:**

The online version contains supplementary material available at 10.1186/s12870-025-06999-5.

## Background

Proso millet (*Panicum miliaceum L.;* 2n = 36) is a C_4_ short-day crop with many superior agronomic traits, such as increased water-use efficiency, a low respiration rate, and a shorter life cycle (60–90 days). It is well adapted to extreme environmental conditions [1], making it a desirable crop for semiarid regions. It is widely used in dryland farming or as a summer rotation crop in temperate regions [2]. Consequently, the breeding of proso millet with improved productivity is highly important, but efforts through conventional breeding have shown slow progress [3]. The rapid development of molecular biology and sequencing technologies provides various tools for crop breeding and has been shown to increase the efficiency of breeding efforts in other cereal crops [3]. On the other hand, proso millet remains an underutilized and unexploited crop partially because of the limited availability of genomic resources [4]. The release of the assembled proso millet genome has made genetic and genomic studies easier, similar to those of other crops [5], which is essential for designing effective regional breeding programs. Nevertheless, this type of information remains limited or is almost entirely lacking in regional proso millet breeding materials.

In green plants, chloroplasts (cp.) are essential for photosynthesis [6]. It is a complex hierarchical process that assimilates chloroplast carbon and diverts it to other compartments to maintain metabolic activities. Biomass determines growth and development to translate it into grain or seed yield [7]. The cp. genome has a highly conserved quadripartite sequence [8]. This sequence generally consists of a large single-copy (LSC) region, a small single-copy (SSC) region and two inverted repeats (IR) [9]. The cp. genome is characterized by non-meiotic and uniparental inheritance, which is mostly maternal [10]. They are ideal for studying plant phylogeography, genetic diversity, and evolution [11].

C_4_ photosynthesis is a complex pathway that is further subdivided into three subtypes on the basis of the predominant decarboxylating enzymes of the four-carbon acid, NAD-dependent malic enzyme (NAD-ME), NADP-dependent malic enzyme (NADP-ME), and PEP carboxykinase (PEPCK) [12]. Many cereal crops, such as maize and sorghum, fall into the NADP-ME subtype, whereas the NAD-ME subtype comprises switchgrass, pearl millet, and Amaranthus [13]. Proso millet lies in the NAD-ME subgroup [14], which uses aspartate as the transport metabolite in place of malate and hence has a relatively high nitrogen-use efficiency [15]. This makes the photosynthesis mechanism in C_4_ plants distinct from that in C_3_ plants, thereby increasing water-use efficiency. As a result, C_4_ plants are extremely resilient to unfavorable weather conditions and shown relatively higher tolerance to droughts [16]. Several studies have been conducted through cp. genome annotation to obtain clear molecular insight into the mechanism of photosynthesis in C_4_ plants. Cp genomes have been instrumental in clarifying evolutionary connections within phylogenetic groups and revealing significant differences in sequence and structure among various plant species [17]. The unique markers identified between the cp. genomes also hold potential for species identification besides molecular breeding.

Hence, this study was conducted to (i) compare the cp. genome organization of ATL1 and TN*Pm* PEM 001 and determine phylogenetic relationship, (ii) identify the key genes involved in photosynthesis and (iii) identify the unique SSRs present in the cp. genome of regional *P. miliaceum L.* cv. ATL1 and its mutant, TN*Pm*PEM 001. Despite its photosensitivity, ATL1 has traditionally been cultivated under rainfed conditions in southern India,, whereas TN*Pm*PEM 001 is a relatively high-yielding and photo-insensitive line. Recently, the whole-genome sequence of proso millet was reported [18], which was used to compare and annotate the cp. genomes generated in this study. Furthermore [19] and [20], sequenced and assembled the cp. genomes of proso millet. To gain additional knowledge on the cp. genome of native proso millet and translate such knowledge to the regular breeding program, the assembled cp. genome reported by [20] was used as a reference (hereafter denoted as “reference PM”). Marker analysis was also performed to identify potential chloroplast SSR (cpSSR) markers with these cp. genomes for upcoming breeding initiatives that use the studied lines and create climate-resilient cultivars.

## Materials and methods

### Plant materials

**Fig. 1:**
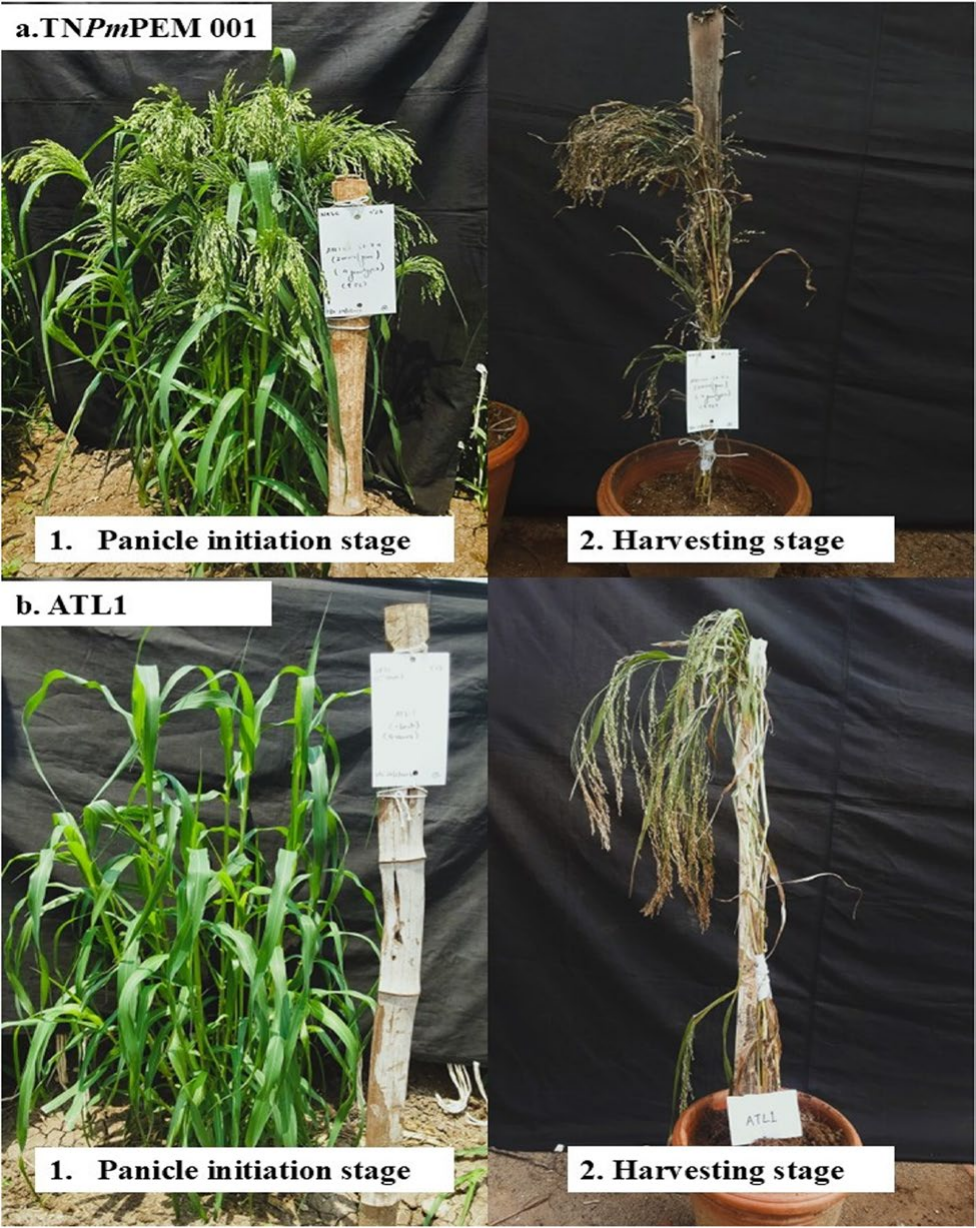
Comparison between **a** TN*Pm*PEM 001 and **b** ATL1 at two different phenological stages: (1) Panicle initiation and (2) Harvesting

ATL1 is medium duration, high-yielding, sturdy proso millet variety released by the Centre of Excellence in Millets, Athiyandal, TNAU, by hybridizing TNAU-164 × IPM-19, followed by selection. It has bold seeds, golden yellow grains and nonlodging characteristics. Induced mutagenesis of ATL1 *via* ethyl methane sulfonate, resulted in a set of mutant lines [21] which were advanced to M_4_ generation through self-pollination. Seeds of these investigated lines are stored in the seed storage unit of the Department of Millets, CPBG, TNAU, Coimbatore, India and they were multiplied and maintained by duly following approved agronomic practices of TNAU. All those M_4_ mutant lines were initially phenotyped *via* the Soil Plant Analysis Development (SPAD), which gives relative chlorophyll content and Portable Photosynthesis System (PPS) to identify a line with a relatively high photosynthetic rate. SPAD and PPS readings showed a positive correlation with the photosynthetic rate [21]. The phenotypic data of the mutant lines were then compared with those of the wild type (hereafter denoted as ATL1) and one mutant line that has shown the highest SPAD and PPS values, early flowering and highest yield per plant was selected (hereafter denoted as TN*Pm*PEM 001) for next-generation sequencing (Fig. 1; Table 1).

**Table 1:** Performance of TN*Pm*PEM 001 and ATL1 under field conditions

Parameter	Days to heading (DAS)	Days to 50% flowering (DAS)	Days to harvesting (DAS)	SPAD reading	PPS reading			Yield/plant (gm)
					*Pn*	C	T	
ATL1	35–40	50–55	80–85	46.80	37.185	0.1483	6.4275	15.0
TN*Pm*PEM 001	25–29	35–40	55–60	48.12	30.414	0.1622	5.5679	40.0

*DAS* Days after sowing, *SPAD* Soil plant analysis development, *PPS* Portable photosynthesis system, *Pn* Photosynthesis rate, *C* Stomatal conductance, *T* Transpiration

### DNA extraction and sequencing

The variety ATL1 and its mutant TN*Pm*PEM 001 were grown at the Department of Millets, Centre for Plant Breeding and Genetics, TNAU, Tamil Nadu, India, during the summer season in 2023. The leaf samples were collected and frozen immediately in liquid nitrogen (−196°C (−321°F).) for further DNA isolation. The total genomic DNA was extracted from leaves via the NucleoSpin Tissue Genomic DNA Purification Kit (Macherey-Nagel, USA) according to the manufacturer’s recommendation, and the quality of the DNA was determined via 1% agarose and a Qubit 4.0 fluorometer (Life Technologies, USA). The whole-genome libraries were prepared via the FS Pro DNA Lib Prep Kit for Illumina (ABclonal, USA) according to the instructions provided by the manufacturer, and paired-end (150 PE) sequencing was performed at Oneomics Private Limited, Tiruchirappalli, Tamil Nadu, India, *via* the NovaSeq 6000 platform (Illumina).

### Chloroplast genome assembly and annotation

Approximately 30 Gb of raw data were preprocessed using Trim Galore v0.6.7 (https://github.com/FelixKrueger/TrimGalore), and the chloroplast DNA (cpDNA) was *de nova* assembled using the GetOrganelle v1.7.0 pipeline [22], with K-mer values set to 75, 95, 115 and 127. The organellar genomes of ATL1 and the mutant line TN*Pm*PEM 001 were annotated with CHLOROBOX GeSeq v2.03 (https://chlorobox.mpimp-golm.mpg.de/geseq.html). The search settings included the prediction of tRNA genes with tRNAscan-SE v.2.0.7 (http://trna.ucsc.edu/tRNAscan-SE/) and with ARAGORN v.1.2.38 (http://www.trna.se/ARAGORN/), with the latter having a maximum intron size of 3,000 bp and a genetic code of “bacterial/plant plastid”. The HMMER tool was used for predicting coding DNA sequences (CDSs) and ribosomal RNAs (rRNAs) *via* a reference chloroplast database (https://www.ncbi.nlm.nih.gov/datasets/genome/GCA_003046395.2/). The circular cpDNA map was drawn *via* Organellar Genome DRAW (OGDRAW v1.3.1) (https://chlorobox.mpimp-golm.mpg.de/OGDraw.html).

### Simple sequence repeat (SSR) analysis

SSRs in the cp. genomes of ATL1 and TN*Pm*PEM 001 were analyzed using the MISA software available at http://pgrc.ipk-gatersleben.de/misa [23], with parameters of 1–10, 2–5, 3–5, 4–5, 5–3, and 6–3, such that there were no fewer than ten mononucleotide repeats, no fewer than five dinucleotides, tri- and tetranucleotide repeats, and at least three pentanucleotide and hexanucleotide repeats. The SSR primers were identified with the help of MegaSSR (https://bioinformatics.um6p.ma/MegaSSR/) using default settings [24].

### Homology and relative codon usage analysis

A homology study was conducted between ATL1 and TN*Pm*PEM 001 with the reference PM, little millet and switchgrass. The mVISTA online software (https://genome.lbl.gov/vista/mvista/about.shtml) was used to generate a sequence variation map by keeping the reference PM as a fixed reference and keeping the shuffling LAGAN as an alignment program. Additionally, mauve analysis was performed to check the homology between the given species. Consequently, an analysis was performed for the relative synonymous codon usage values (RSCU) on the basis of gene coding sequences *via* the RSCU calculator (https://jamiemcgowan.ie/bioinf/rscu.html).

### Phylogenetic analysis

Phylogenetic analysis was done to identify the position of newly assembled cp. genome within the PACMAD clade. The complete cp. genome sequences of the Poaceae family, particularly *Panicum* spp., and other crop species were obtained from the NCBI database. Phylogenetic analysis was performed using MAFFT (https://mafft.cbrc.jp/alignment/server/index.html), with a bootstrap value of 1000, using the Maximum Likelihood method where soybean, cotton, tobacco and pea cp. genomes were used as outgroups.

## Results

The investigated plant materials showed that the mutant line, TN*Pm*PEM 001, matured earlier (55–60 days) and produced a higher yield (40 g/plant) than ATL1. This improved performance in TN*Pm*PEM 001 was likely due to its potentially greater photosynthetic capacity (SPAD reading) and increased stomatal conductance resulting in increased growth rate and biomass accumulation (Table 1; Fig. 1).

### Chloroplast genome assembly of ATL1 and TN*Pm*PEM 001

**Fig. 2:**
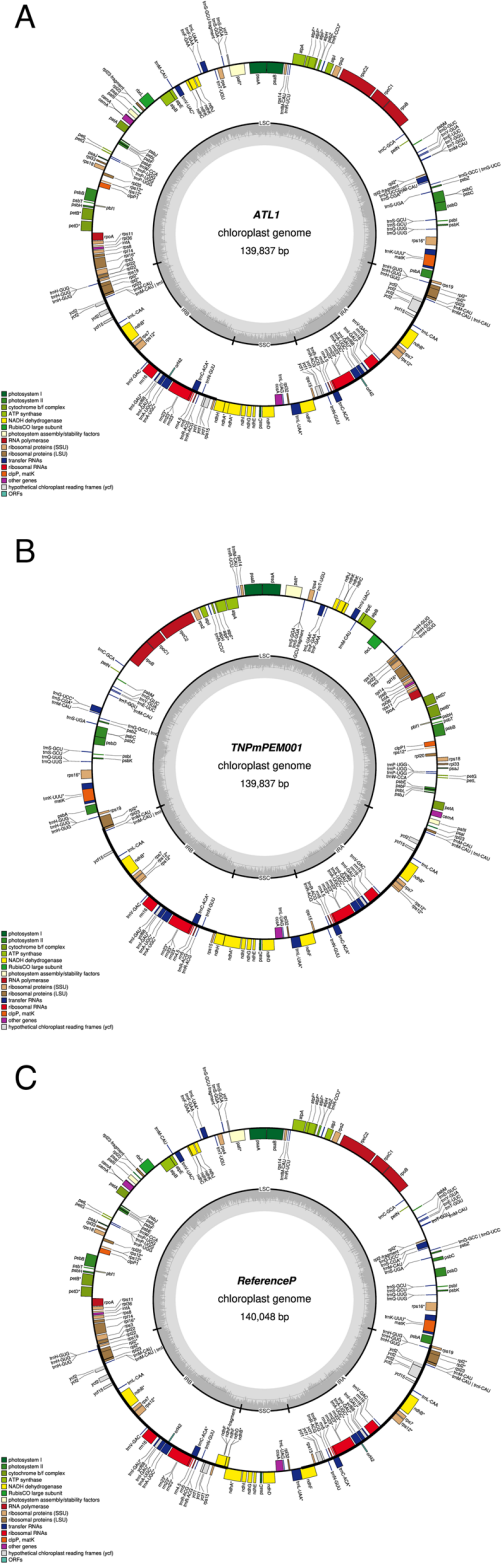
Cp genome map of **a** ATL1 **b** TN*Pm*PEM 001 and **c** reference PM. The genes located on the outer side of the circle are in the clockwise direction, and the genes present in the inner circle are in the anticlockwise direction. The different color coding represents genes with different functions. Pink arrow mark indicates both the starting point and progressing direction of gene annotation

The cp. genomes of ATL1 (SRX25923566) and its mutant TN*Pm*PEM 001 (SRX25923567) (BioProject accession number: PRJNA1154795 available at https://www.ncbi.nlm.nih.gov/sra/PRJNA1154795) assembled in this study were compared with the already published cp. genome of the reference PM [20]. The annotated cp. genomes of TN*Pm*PEM 001 and ATL1, both 139 837 bp in length, presented a typical quadripartite structure, consistent with the reference PM genome (139 826 bp) (Table 2; Fig. 2). The cp. genome of ATL1 consists of 42 502 bp of A (30.310%), 43 344 bp of T (30.994%), 27 242 bp of G (19.481%) and 26 749 bp (19.128%) of C, whereas TN*Pm*PEM 001 consists of 41 468 bp (29.654%) of A, 43 344 bp (30.996%) of T, 27 132 bp (19.402) of G and 26 859 bp (19.207%) of C (data not shown).

**Table 2:** Comparison of the cp. genome features of ATL1 and TN*Pm*PEM 001 with those of the reference PM and other millets

Particulars	Reference PM	ATL1	TNPmPEM 001	Sorghum	Pearl millet	Little millet	Finger millet	Barnyard millet	Foxtail millet
Total length (bp)	139,826	139,837	139,837	140,754	138,172	139,384	135,137	139,593	135,516
SSC length (bp	12,574	12,795	12,795	12,503	12,409	12,583	12,636	12,518	12,012
LSC length (bp)	81,682	84,522	84,522	82,685	81,213	81,355	80,663	81,839	79,896
IR length (bp)	22,785	20,560	20,560	22,783	22,275	22,723	41,838	22,618	21,804
Genes	108	111	111	110	110	125	108	112	111
CDS	76	77	77	77	76	91	76	77	71
tRNAs	28	30	30	29	30	30	28	30	36
rRNAs	4	4	4	4	4	4	4	4	4
GC content (%)	38.6	38.6	38.6	38.50	38.6	38.6	38.13	38.6	38.87
Reference	[20]	Current study	Current study	[25]	[26]	[27]	[28]	[29]	[30]

*LSC* Large single-copy region, *SSC* Small single-copy region, *IR* Inverted repeats, *CDS* Coding DNA sequence, *tRNA* Transfer RNA, *rRNA* Ribosomal RNA

Furthermore, ATL1 and TN*Pm*PEM 001 had a total of 38.6 GC%, similar to the reference PM’s cp. genome. Compared with the cp. genomes of the other millet varieties, the investigated cp. genomes were slightly larger than those of little millet (139 384 bp), barnyard millet (139 593 bp), pearl millet (138 172 bp), finger millet (135 137 bp) and foxtail millet (135 516 bp). However, the sorghum cp. genome length (140 754 bp) was greater than those of ATL1 and TN*Pm*PEM 001 (Table 2).

Both the ATL1 and TN*Pm*PEM 001 cp. genome assemblies displayed a quadripartite structure, characterized by a LSC of 84 522 bp, and a conserved SSC of 12 795 bp. These were flanked by inverted repeat regions (IRA and IRB) of 20 560 bp in both the genome (Table 2; Fig. 2).

The list of annotated genes and their functions investigated in this study is provided in Supplementary Table 1. There were 57 photosynthesis-related genes (ATP synthase, Photosystems I and II, the cytochrome b/f complex, NADH dehydrogenase and Rubisco) and 22 self-replicating genes (controlling the large and small subunits of the ribosome and DNA-dependent RNA polymerase).

The number of rRNA genes (4) was equal in ATL1 and TN*Pm*PEM 001 as that of reference PM (Table 2). However, TN*Pm*PEM 001 and ATL1 had similar numbers of tRNA genes (30), which was slightly greater (28) than that of the reference PM (Table 2). The list of tRNA genes that were common to both ATL 1 and TN*Pm*PEM 001 when compared with the reference PM is provided in Supplementary Table 2.

Further analysis revealed that the cp. genomes of both ATL1 and TN*Pm*PEM 001 consisted of 111 genes, which was greater than the total number of genes (108) found in the reference PM (Table 2). Among the 111 genes, 77 were coding DNA sequences (CDS; protein-coding genes), whereas they were 76 in the reference PM. Interestingly, they were not reflecting the same set of genes. For example, *psbN*,* ycf3a*,* ycf4*,* rplc*,* rpl23c*,* rps7c*,* rpsc*,* rpsd*,* rps15c*,* ycf15c*,* ycf68c* and *orf42* genes were present only in reference PM but absent in ATL1 and TN*Pm*PEM 001. On the other hand, *atpF*,* rpl23*,* rps15*,* pvf1*,* ycf2*,* pafI* and *pafII* genes were found both in ATL1 and TN*Pm*PEM 001 but absent in reference PM (Table 3).

**Table 3:** Genes identified in the cp genomes of reference PM, ATL1 and TN*Pm*PEM 001

				**Presence/absence of gene **		
	Category	Group of genes	Name of genes	Reference PM	ATL1	TN*Pm*PEM 001
1			*atpA*	+	+	+
2			*atpB*	+	+	+
3		ATP synthase	*atpE*	+	+	+
4			*atpH*	+	+	+
5			*atpI*	+	+	+
6			*atpF*	-	++	++
7			*psbA*	+	+	+
8			*psbB*	+	+	+
9			*psbC*	+	+	+
10			*psbD*	+	+	+
11		Photosystem II	*psbE*	+	+	+
12			*psbF*	+	+	+
13			*psbH*	+	+	+
14			*psbI*	+	+	+
15			*psbJ*	+	+	+
16			*psbK*	+	+	+
17			*psbL*	+	+	+
18			*psbM*	+	+	+
19			*psbN*	+	-	-
20			*psbT*	+	+	+
21			*psbZ*	+	+	+
22	Gene for photosynthesis		*ndhA*	+	++	++
23			*ndhB*	+	++++	++++
24			*ndhC*	+	+	+
25			*ndhD*	+	+	+
26		NADH-dehydrogenase	*ndhE*	+	+	+
27			*ndhF*	+	+	+
28			*ndhG*	+	+	+
29			*ndhH*	+	+	+
30			*ndhI*	+	+	+
31			*ndhJ*	+	+	+
32			*ndhK*	+	+	+
33			*petA*	+	+	+
34			*petB*	+	++	++
35		Cytochrome b/f complex	*petD*	+	++	++
36			*petG*	+	+	+
37			*petL*	+	+	+
38			*petN*	+	+	+
39			*psaA*	+	+	+
40			*psaB*	+	+	+
41		Photosystem I	*psaC*	+	+	+
42			*psaI*	+	+	+
43			*psaJ*	+	+	+
44			*ycf3a*	+	-	-
45			*ycf4*	+	-	-
46		Rubisco	*rbcL*	+	+	+
47			*rpl2*	+	++++	++
48			*Rplc*	+	-	-
49			*rpl14*	+	+	+
50			*rpl16*	+	++	++
51		Large subunit of ribosome	*rpl20*	+	+	+
52			*rpl22*	+	+	+
53			*rpl23*	-	++	++
54			*rpl23c*	+	-	-
55			*rpl32*	+	+	+
56			*rpl33*	+	+	+
57			*rpl36*	+	+	+
58			*rps2*	+	+	+
59			*rps3*	+	+	+
60			*rps4*	+	+	+
61			*rps7*	-	++	++
62			*rps7c*	+	-	-
63			*rps8*	+	+	+
64			*rps11*	+	+	+
65		Small subunit of ribosome	*rps12*	+	+++++	+++++
66			*rpsc*	+	-	-
67	Self-replication		*rpsd*	+	-	-
68			*rps14*	+	+	+
69			*rps15*	-	++	++
70			*rps15c*	+	-	-
71			*rps16*	+	++	++
72			*rps18*	+	+	+
73			*rps19*	+	++	+
74			*rpoA*	+	+	+
75		DNA dependent RNA polymerase	*rpoB*	+	+	+
76			*rpoC1*	+	++	++
77			*rpoC2*	+	+	+
78		C-type cytochrome synthesis gene	*ccsA*	+	+	+
79		Envelop membrane protein	*cemA*	+	+	+
80		Protease	*clpP*	+	+++	+++
81	Other genes	Translational initiation factor	*infA*	+	+	+
82		Maturase	*matK*	+	+	+
83	Unknown genes		*pbf1*	-	+	+
84			*ycf2*	-	+	+
85			*pafI*	-	+++	+++
86			*pafII*	-	+	+
87			*ycf15c*	+	-	-
88			*ycf68c*	+	-	-
89			*orf42*	+	-	-
Total				76	77	77

*the “+” sign represents the presence of gene and the "-" sign represents the absence of gene. additional/multiple “+” sign represents presence of multiple copies (‘n’ numbers) of the concerned gene

### Comparison of the assembled chloroplast genome and the genomes of other millet chloroplasts

The assembled cp. genomes of ATL1 and TN*Pm*PEM 001 were also compared with the published cp. genomes of other millets. Although the assembled cp. genomes were larger than that of foxtail and finger millet, both assembled genomes have cp. genome sizes that are comparable to those of little millet. The SSC, LSC, and IR were found to be almost similar in length in all the investigated millets compared with ATL1 and TN*Pm*PEM 001 (Table 2). Among all the compared cp. genomes, little millet has the greatest number of coding DNA sequences (91), whereas *Setaria italica* has only 71 coding DNA sequences (Table 2).

The CPGview [31] of the cp. genome revealed important information of cis-splicing genes in ATL1 and TN*Pm*PEM 001, that play important roles in photosynthesis (data not shown).

### Simple sequence repeat (SSR) analysis

**Fig. 3:**
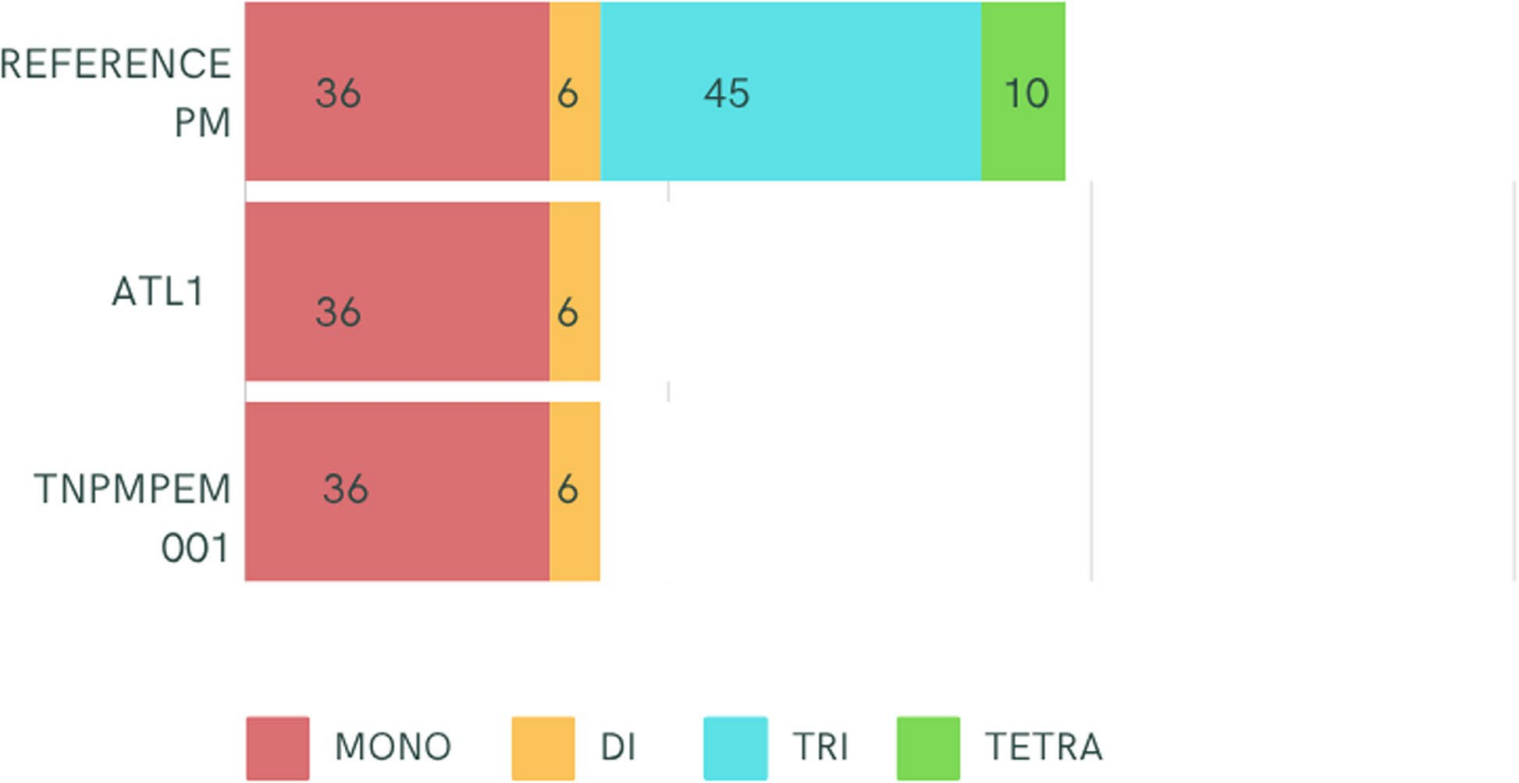
Distribution of different simple sequence repeat classes in the cp. genomes of the reference PM, ATL1 and TN*Pm*PEM 001

Several SSRs were identified in the cp. genomes of ATL1 and TN*Pm*PEM 001 (Table 4). Those SSRs were then compared with the already identified SSRs from the reference PM [20], which also employed the same parameters that were used in this study for identifying SSRs. It has been found that tri- and tetra- repeat motifs were found in reference PM but absent in ATL1 and TN*Pm*PEM 001 (Fig. 3). A total of 42 SSRs were identified in ATL1 and TN*Pm*PEM 001, comprising 36 monomeric and 6 dimeric repeats (Fig. 3).

**Table 4:** List of identified unique SSRs in ATL1 and TNPmPEM 001

SSR Code	Position (bp)(Start-End)		SSR motif		Repeat size		Melting temperature		Forward primer	Reverse primer
	ATL1	TN ***Pm*** PEM 001	ATL1	TN *Pm* PEM 001	ATL1	TN *Pm* PEM 001	Forward	Reverse		
Pm-CpSSR 001	12,547–12,888	47,834–48,176	T	T	14	15	59.818	59.964	TCCTTTGCACCCGACTCAAA	ACCTGTTTTCGGCCCTACAG
Pm-CpSSR 002	66,668–67,155	-	(TA)_5_gtatatgaatcaaataatatatggatcaaagaaagactacttcttctggatccaaaattaataaaataaagaaatcca(T)_12_	-	100	-	58.497	59.89	TGTCGTTATTGTCGCAAGCA	AATCAATTCGATCCCCCGGG
Pm-CpSSR 003	77,689–78,003	-	(T)_10_ctctccta(T)_10_	-	28	-	60.179	60.105	ATACGCCCCTTGCTGGAATC	TGGCCCCACCTATAGTACCC
Pm-CpSSR 004	79,843–80,191	-	(T)_11_attttattatattgatgctttatcacattgc(T)_10_	-	52	-	59.97	59.598	GGTTTTGTCGTTCCCACAGC	CCGGATTTTAGGCTGTGAAGC
Pm-CpSSR 005	65,187–65,437	-	T	-	11	-	59.418	60.038	GCGGGATTATTCGTGACTGC	TTTGGTAGAACGCGGGTCTC
Pm-CpSSR 006	65,857–66,111	-	TA	-	10	-	57.251	60.542	AGGAAGAAGGGGTCATCTTTTT	CCTGCTAAAGCCCCAAACCA
Pm-CpSSR 007	75,493–75,756	-	A	-	11	-	60.414	57.395	TTGATTCATTCAACCGCGAAGC	TGGGACACTCTAGAGAAGCA
Pm-CpSSR 008	79,495–79,806	-	T	-	12	-	60.249	60.325	CTTCTCATCCAGCTCCTCGC	ACTTCATTGGGTGGGATGGC
Pm-CpSSR 009	80,290–80,587	-	T	-	10	-	59.312	60.064	AGGATCTCGACAATACGAAGCA	CCCTGTCTTCCCATTCTTCCC
Pm-CpSSR 010	81,188–81,489	-	T	-	10	-	59.542	59.998	TGGTTGTGCGAACCAAAAGG	AGGGATTTCGACAAAGCGAGT
Pm-CpSSR 011	104,784–105,044	-	AG	-	10	-	58.914	59.968	AGCAATAACGGTAAATGCACCA	CAGCAGCTACTCCTCCGATG
Pm-CpSSR 012	106,759–107,078	-	A	-	10	-	59.757	58.529	TCCGGTCTAGAGTATGTTCCCA	TGCTTCCGAATTGATCTCATCC
Pm-CpSSR 013	-	10,036–10,355	-	T	-	10	58.529	59.757	TGCTTCCGAATTGATCTCATCC	TCCGGTCTAGAGTATGTTCCCA
Pm-CpSSR 014	-	12,070–12,330	-	TC	-	10	59.968	58.914	CAGCAGCTACTCCTCCGATG	AGCAATAACGGTAAATGCACCA
Pm-CpSSR 015	-	95,937–96,238	-	A	-	10	59.998	59.542	AGGGATTTCGACAAAGCGAGT	TGGTTGTGCGAACCAAAAGG
Pm-CpSSR 016	-	96,839–97,136	-	A	-	10	60.064	59.312	CCCTGTCTTCCCATTCTTCCC	AGGATCTCGACAATACGAAGCA
Pm-CpSSR 017	-	97,235–97,583	-	(A)_10_gcaatgtgataaagcatcaatataataaaat(A)_11_	-	52	59.598	59.97	CCGGATTTTAGGCTGTGAAGC	GGTTTTGTCGTTCCCACAGC
Pm-CpSSR 018	-	97,620–97,931	-	A	-	12	60.325	60.249	ACTTCATTGGGTGGGATGGC	CTTCTCATCCAGCTCCTCGC
Pm-CpSSR 019	-	99,423–99,737	-	(A)_10_taggagag(A)_10_	-	28	60.105	60.179	TGGCCCCACCTATAGTACCC	ATACGCCCCTTGCTGGAATC
Pm-CpSSR 021	-	101,670–101,933	-	T	-	11	57.395	60.414	TGGGACACTCTAGAGAAGCA	TTGATTCATTCAACCGCGAAGC
Pm-CpSSR 021	-	110,271–110,758	-	(A)_12_tggatttctttattttattaattttggatccagaagaagtagtctttctttgatccatatattatttgattcatatac (TA)_5_	-	100	59.89	58.479	AATCAATTCGATCCCCCGGG	TGTCGTTATTGTCGCAAGCA
Pm-CpSSR 022	-	111,315–111,569	-	TA	-	10	60.542	57.251	CCTGCTAAAGCCCCAAACCA	AGGAAGAAGGGGTCATCTTTTT
Pm-CpSSR 023	-	111,989–112,239	-	A	-	11	60.038	59.418	TTTGGTAGAACGCGGGTCTC	GCGGGATTATTCGTGACTGC

Although a similar number of SSRs were identified between ATL1 and TN*Pm*PEM 001, few were polymorphic (i.e., there were differences in SSR motif repeats; Table 4). Among them, the frequencies of A and T in the SSR repeat motifs that differentiate ATL1 and TN*Pm*PEM 001 were the highest (Supplementary Table 3).

Primers were also designed for the identified common SSR motifs (including 24 mono-, 5 di- and 4 compound repeats and described in Table 4 and Supplementary Table 4. Among these SSRs, 11 SSRs were exclusively found in ATL1; similarly, another set of 11 SSRs was unique to TN*Pm*PEM 001 (Table 4). Interestingly, one SSR (Pm-CpSSR 001) was shown to have a polymorphic mono-repeat motif in the two investigated samples: 14 repeats in ATL1 and 15 repeats in TN*Pm*PEM 001 (Table 4). It was also inferred that the minimum repeat size for the identified SSRs was 10, whereas the maximum repeat size was 100 for a single SSR motif in the two investigated lines.

### Codon usage pattern

Relative synonymous codon usage (a ratio between the observed frequency of a codon to the expected frequency of all synonymous codons used for an amino acid) is a statistical index used to weigh the relative frequency of each synonymous codon. The cp. genomes of ATL1 and TN*Pm*PEM 001 include 64 codons. In addition to three stop codons, 32 equal numbers of codons ending with A/T and G/C were identified in both samples. In ATL1, 30 codons had relative synonymous codon usage (RSCU) values greater than 1, whereas codons for tryptophan and methionine had RSCU values equal to 1 in both cases, indicating a lack of bias for the codon (Supplementary Table 5). For TN*Pm*PEM 001, 31 codons have values greater than 1, indicating that the codon is biased and is used more frequently. In both cases, the codons with higher RSCU values have A/T as their third base, indicating that the cp. genomes of ATL1 and TN*Pm*PEM 001 prefer codons ending with A or T.

### Homology analysis

**Fig. 4:**
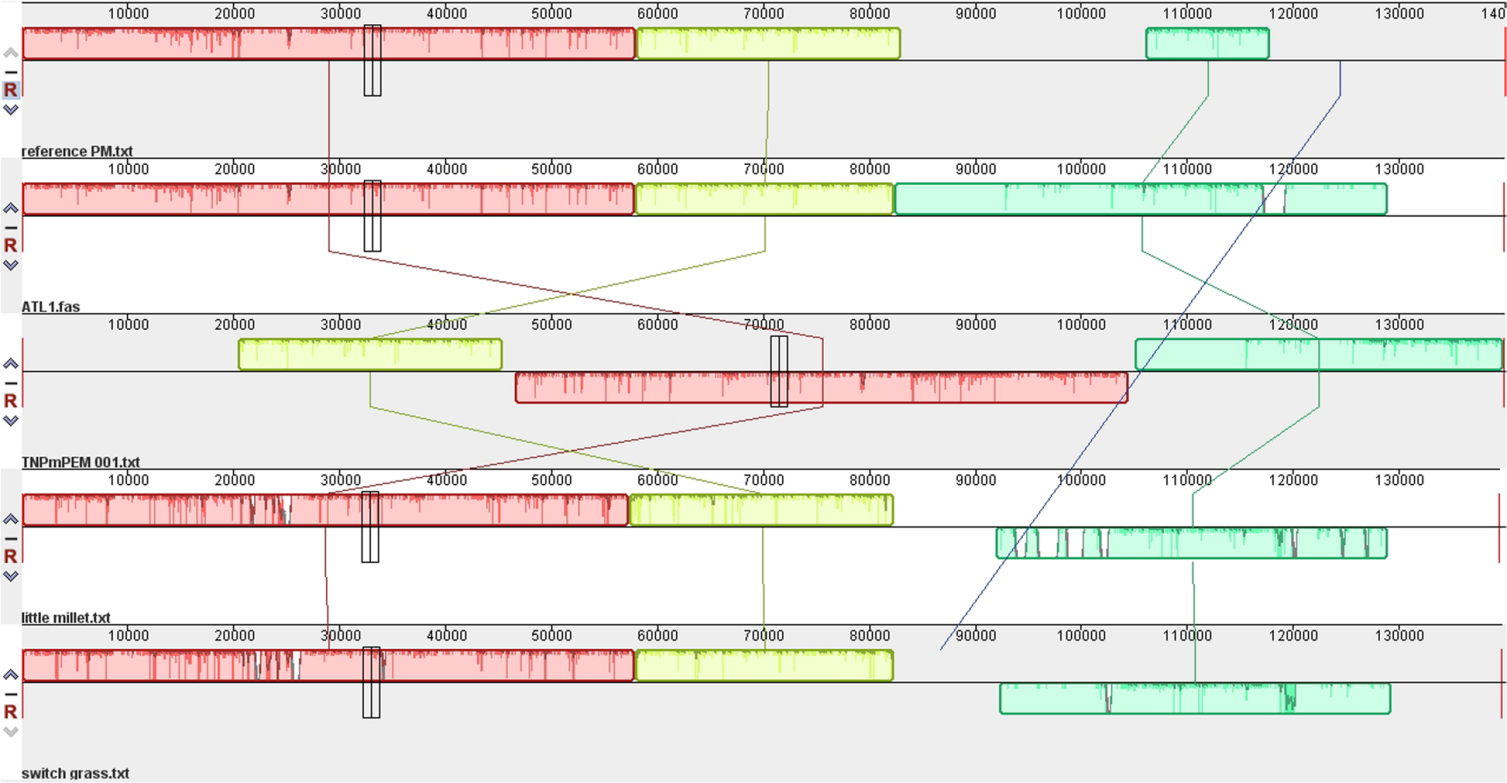
Mauve synteny analysis among Reference PM, ATL1, TN*Pm*PEM 001, Little millet and Switchgrass

**Fig. 5:**
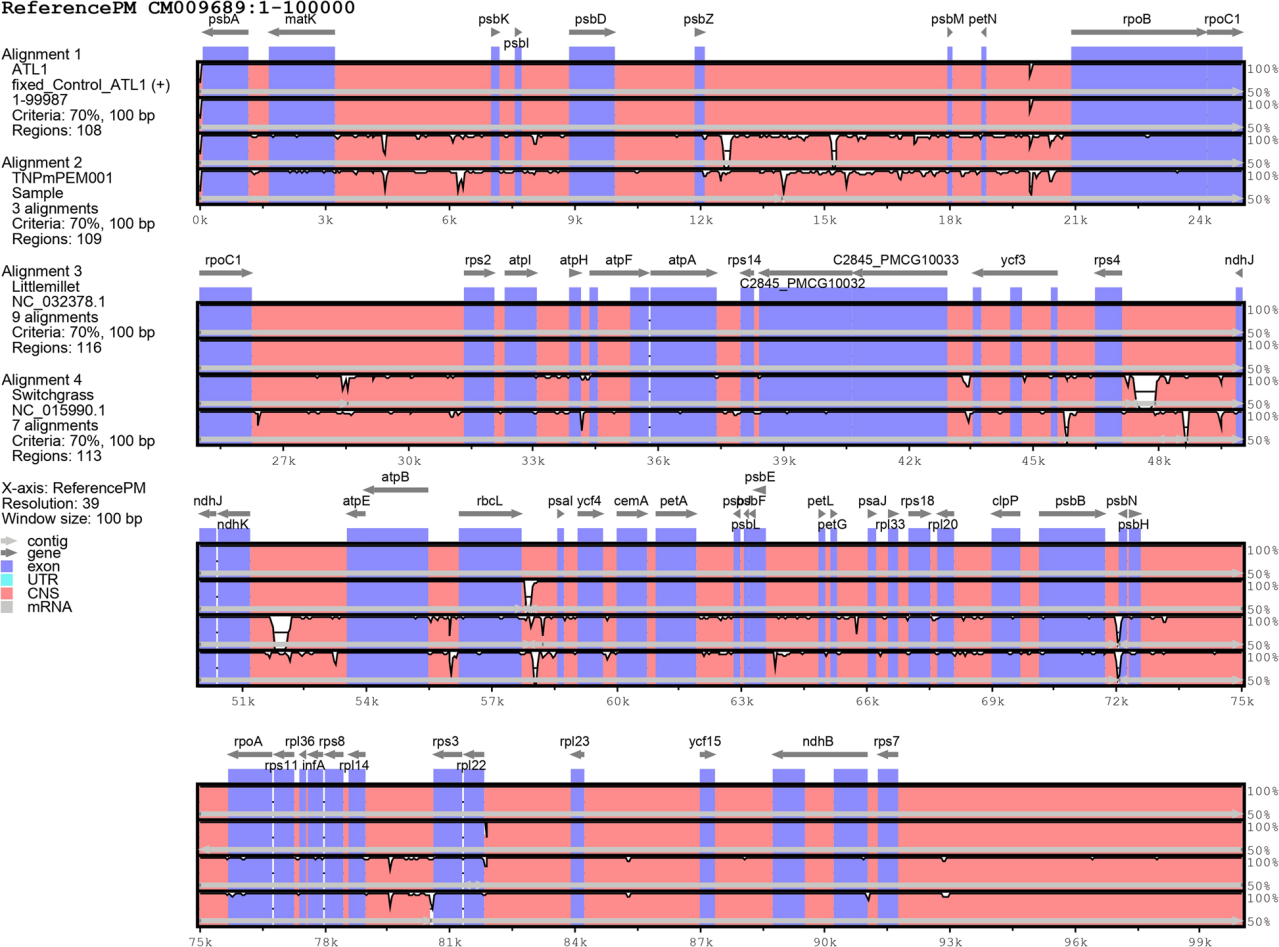
mVISTA analysis of the chloroplast sequence of (1) ATL1, (2) TN*Pm*PEM 001, (3) Little millet, (4) Switchgrass clusters with reference PM

To gain further insight into the cp. genomes of ATL1 and TN*Pm*PEM 001, a homology study was performed *via* mauve and mVISTA software to assess the alignment and rearrangement between the annotated species and closely related crop species, such as little millet and switchgrass, which are generally closely associated with proso millet as described elsewhere [32]. In all four sequences, namely ATL1, the reference PM, little millet and switchgrass, the locally collinear block (LCB) was at a similar location, showing a conserved arrangement, whereas TN*Pm*PEM 001 rearranged in the LCB, suggesting possible rearrangements due to inversion or translocation (Fig. 4). The vertical white gaps in the LCB depict the probability of horizontal gene transfer or gene loss in a specific genome. The differences between ATL1 and TN*Pm*PEM 001 and other species were analyzed *via* mVISTA. The results revealed that the sequences were highly similar to each other. In general, protein-coding regions presented high levels of conservation in all five species. Low similarity can be observed in the noncoding regions of ATL1, TN*Pm*PEM 001 and little millet, suggesting greater variability (Fig. 5).

### Phylogenetic analysis

**Fig. 6:**
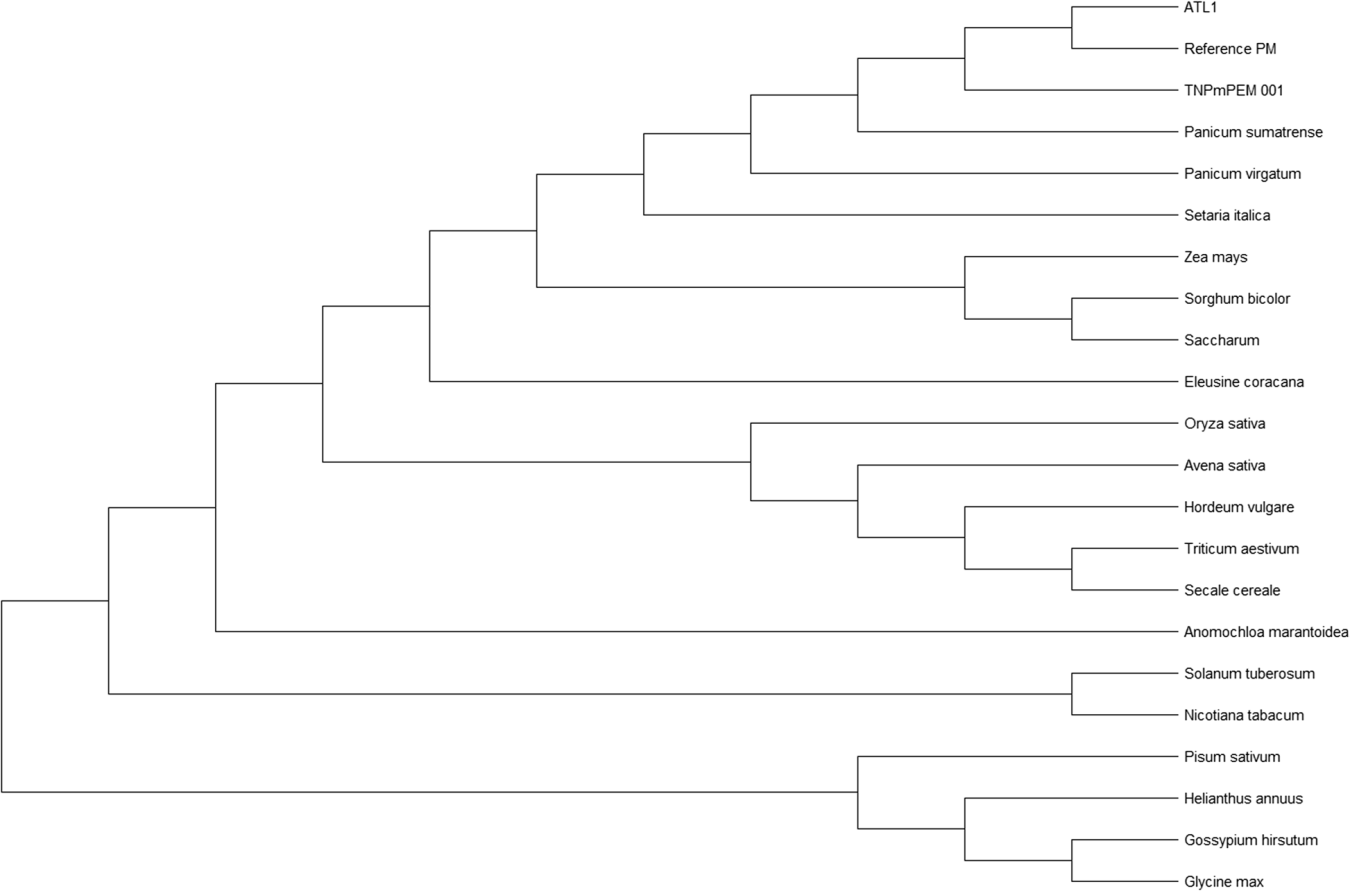
Phylogenetic tree constructed via maximum likelihood (ML) based on chloroplast-genome sequences of ATL1, TN*Pm*PEM 001, Proso millet (reference PM (CM009689 *Panicum miliaceum*), Maize (KF241981 *Zea mays*), Rice (NC_031333 *Oryza sativa*), Wheat (NC_002762 *Triticum aestivum*), Foxtail millet (KF646538 *Setaria italica*), Sorghum (EF115542 *Sorghum bicolor*), Barley (NC_056985 *Hordeum vulgare*), Oat (MK336398 *Avena sativa*), Switchgrass (NC_015990 *Panicum virgatum*), Finger millet (MW080648 *Eleusine coracana*), Rye (NC_021761 *Secale cereale*), Sugarcane (KU214867 *Saccharum officinarum*), Cotton (MG800784 *Gossypium hirsutum*), Soybean (NC_007942 *Glycine max*), Potato (NC_008096 *Solanum tuberosum*), Sunflower (NC_007977 *Helianthus annuus*), Tobacco (Z00044 *Nicotiana tabacum*), *Anomochloa marantoidea* (NC_014062), Pea, (NC_007942 *Pisum sativum*)

A phylogenetic study of the cp. genomes of ATL1 and TN*Pm*PEM 001 was performed to understand the genome evolution, position and relationship with other millet and other staple crop species within and outside the Poaceae family. This tree was broadly divided into 2 major clades and further subdivided into 11 subclasses and as expected the outgroups viz., soybean, cotton, sunflower, and pea were clustered separately. All the members of the Panicoideae subfamily (Paniceae tribe) were clustered together (including ATL1 and TN*Pm*PEM 001), forming a single clade with maize, sorghum and sugarcane (Fig. 6). As finger millet (*Eleusine coracana*) belongs to the Chloridoideae subfamily (Eragrostideae tribe) (https://themilletproject.org/millet-taxonomy/), it was placed in a different clade than rice and wheat. Furthermore, this study revealed a direct relationship between the assembled cp. genome and *P. sumatrense* at 100 bootstrap values, which revealed an evolutionary link between them.

## Discussion

In general, cp. genome sequencing is considered a cutting-edge tool for phylogenetic analysis [19], and it can provide a rich source of nucleotide and amino acid sequence data that can be used to address phylogenetic and molecular evolutionary questions [34]. The South Indian proso millet variety, ATL1 was used in this study to create a photo-insensitive and early flowering mutant line. Since SPAD and PPS-derived data support increased yield [35], initially these lines were evaluated for these readings. The relationship between earliness and photo-insensitivity in cultivars has been extensively studied in maize [36] and pearl millet [37] and concluded that photo insensitivity and earliness were directly related [38, 39]. This study also confirmed this relationship (Table 1).

### Chloroplast assembly of ATL1 and TN*Pm*PEM 001

The cp. genome provides rich information for studying comparative genome evaluation, phylogenetics, population genetics and genetic transformation [40]. The cp. genome of the Poaceae family is approximately 125–165 kb in size and can be divided into four parts: the LSC, SSC and two IR regions, with sizes ranging from 75 to 87 kb, 12–14 kb, and 18–32 kb, respectively [41]. The assembled cp. genomes reported in this study also supported the criteria for the cp. genome of the Poaceae family (Table 2). Furthermore, the chloroplast genes were highly similar to those in the cp. genomes of other millet species. This typical quadripartite assembly of the cp. genome indicated that the assembled genome was highly conserved, with identical genes and gene orders [26]. A size difference between the genome assemblies of finger millet and ATL1 and TN*Pm*PEM 001 was also observed, it might be due to the variable size of the inverted repeat regions [42]. The GC content of the assembled genomes was 38.6% and they were within the described GC content range (37–40%) of Poaceae [43, 44].

### Genes identified in the chloroplast genomes of ATL1 and TN*Pm*PEM 001

In this study, genes involved in photosynthesis were identified and they were compared with those of the reference PM. Interestingly, the number of photosynthesis-related genes was the same between ATL1 and TN*Pm*PEM 001, although some differences were observed in the photosynthesis genes present in the reference PM. This difference in number of genes might be due to the rearrangement of the chloroplast genes or the loss of those genes during evolution. A high incidence of gene loss in the algal cp. genome compared with the green alga *Chlamydomonas* has been reported [45, 46]. It has been reported that only forty-four of the 274 plastid-encoded genes are retained in plastid genomes and approximately half of the genes that have been either lost or transferred to the nucleus were missing [47]. The significant difference between the reference PM cp. genome assembly and ATL1 and TN*Pm*PEM 001 might be due to the origin or race of the crop, as the reference PM was the Chinese proso millet cultivar, whereas ATL1 was the variety developed from the Indian proso millet germplasm. In another study [48], it was reported that during cp. genome evolution, the Poaceae family lost three genes viz., *AccD*,* ycf1* and *ycf2*. Thus, it was inferred that loss of genes among different ecotypes would be a general phenomenon during evolution under different environments.

Thus, it can be inferred that genome organization was conserved among all the investigated millet species except for *Eleusine coracana* (Table 2). This variation also supported phylogenesis (Fig. 6), where *Eleusine coracana* (belonging to the Eragrostideae tribe; Chloridoideae subfamily), was different from the other small millet cultivars belonging to the Paniceae tribe (https://themilletproject.org/millet-taxonomy/).

### Analysis of repeat sequences and homologies in the cp. genomes of ATL1 and TN*Pm*PEM 001

It is believed that cpSSR plays an important role in cp. genome rearrangement during evolution [49]; therefore, SSRs identified in the cp. genome can provide useful genetic information and sequence resources for molecular genetic studies in the Poaceae family. Using MISA software, an earlier study found 97 perfect SSRs in the reference PMs’ cp. genome [20]. Nevertheless, in the present study, 42 perfect SSRs were identified. On the other hand, both studies identified prevalent and identical numbers of mononucleotide repeats in SSR loci. These SSRs provide genetic information and would be suitable as markers because of their locus specificity, codominance and highly polymorphic nature, making them useful for studying phylogenetic relationships [50].

The RSCU was analyzed because of the degeneracy of codons. A codon value greater than 1 indicates that the codon has a positive bias and is used more frequently, whereas a codon value less than one indicates that the codon is negatively biased and is used less frequently. If the RSCU value is equal to 1, the codon lacks bias, and codon usage is random [51]. The codon utilization rate varies from species to species. RSCU values are thought to be the result of natural selection, mutation and genetic drift [52]. A total of 30 and 31 codons had RSCU values greater than 1 in ATL1 and TNPmPEM 001, respectively, indicating that these codons were biased or used more than the other codons. The codon encoding arginine (AGA) in TN*Pm*PEM 001 was the most commonly used codon, whereas the codon encoding arginine in CGC was the least commonly used codon. Codons with RSCU values greater than 1 have A/T as the third base, indicating the preference of proso millet for the codon ending with an A or T. Homology analysis *via* mauve software revealed the conserved alignment between the reference PM, ATL1, little millet and switchgrass, whereas rearrangement can be seen in TN*Pm*PEM 001, indicating genomic variation or structural variation. A more similar LCB indicates that genomes are closely related, but rearrangement or inversion is attributed to evolutionary changes [52]. Using the mVISTA analysis, it was found that the coding sequences were highly similar, with high peaks in all the investigated samples showing conserved evolutionary segments. Regions with low peaks reflect low similarity, suggesting that variations between species can be subjected to evolutionary changes [33]. In general, highly conserved regions are functionally significant areas that encode genes or regulatory elements and preserve protein function and gene control during many evolutionary periods.

### Phylogenetic analysis of ATL1 and TN*Pm*PEM 001

The present study also provided insight into the phylogenetic position of the assembled genomes of ATL1 and TN*Pm*PEM 001 with respect to other members of the Poaceae family. In this study, we compared the newly assembled cp. genome with 19 members from different plant families. The results revealed that the assembled cp. genome was a sister to *P. sumatrense*, with the highest bootstrap. Nevertheless, they were distantly connected to *E. coracana* despite belonging to the minor millets. A similar result was reported by [27], who conducted a phylogenetic study for *P. sumatrense* with other Poaceae members and revealed that *P. sumatrense* clustered closely and directly with *P. miliaceum*. Other studies [11] and [32], have also confirmed these relationships. Therefore, the findings of this study indicated that *P. miliaceum* and *P. sumatrense* were sister species. The PACMAD clade consists mainly of C_4_ plants of the grass family. This study revealed that all the C_4_ Poaceae members, including ATL1 and TN*Pm*PEM 001, were clustered in the PACMAD clade (with maize, sorghum and sugarcane). Within the Poaceae family, a distinct sister relationship was revealed between the assembled genomes, reference PM and *P. sumatrense*. This study, supported the findings of [20], who reported a close relationship between *P. miliaceum* and *P. virgatum*, reflecting the convergence in evolutionary relationships at the taxonomic level. The monophyletic relationship of *S. officinarum* also supported the result reported by [20], who described a direct and sister relationship between *S. bicolor* and *S. officinarum*, which also belong to different subtribes: Sorghinase and Saccharinae, respectively.

All of these relationships suggest that it is essential to clarify the evolutionary, phylogenetic and taxonomic relationships of the lower taxon species of the Poaceae family and to perform a large-scale investigation of the diverse Poaceae family to obtain precise and actual classification and evolutionary relationships. A grasp of the Poaceae cp. genome can lead to the development of novel approaches to explore species and for sustainable agriculture.

The cp. genome has been an important source of data for green plant phylogenetic reconstruction [53, 54]. These newly assembled cp. genomes support evolutionary and phylogenetic studies of Indian proso millet (*Panicum miliaceum L.)* in the Poaceae family. Additional research on Indian proso millet at genome level will reveal its molecular mechanism, gene order and arrangement, and relationships with other millet species. As proso millet has a narrow genetic base, new genes for a short life cycle, high photosynthetic efficiency, high yield and pest and disease resistance are needed to increase yield potential and ensure sustainable agriculture. Research in this direction is in progress at the authors laboratory.

## Conclusion

The chloroplast genome characterization of ATL1 and its mutant TNPmPEM 001 provides 11 unique SSR markers for marker-assisted selection in proso millet breeding. These markers can facilitate the tracking of the mutant’s chloroplast, potentially linked to enhanced photosynthetic efficiency or photo-insensitivity. This allows for efficient early-stage selection in breeding programs. The study also lays the groundwork for comparative chloroplast genomics to identify further markers for yield and abiotic stress tolerance. While informative, the chloroplast genome represents only a small window into the plant’s complete genetic picture. Hence, future research should encompass nuclear and mitochondrial genome analyses for a comprehensive understanding of phenotypic variation. While offering valuable initial tools, further validation of marker-trait associations and broader genomic investigations are crucial for impactful proso millet improvement. The authors’ institution is actively engaged in research along these lines.

## Supplementary Information

Supplementary Material 1..

## Data Availability

The cp. genome assembly can be found in GenBank with the accession numbers SRX25923567 for TNPmPEM 001 and SRX25923566 for ATL1. The datasets generated and/or analysed during the current study are available in the BioProject accession number: PRJNA1154795, which can be accessed at https://www.ncbi.nlm.nih.gov/sra/PRJNA1154795. The raw sequencing data were deposited into the NCBI Short Read Archive with the accession number PRJNA1154795.Seed materials mentioned in this report can be obtained from Center for Plant Breeding and Genetics, Tamil Nadu Agricultural University, Coimbatore, India by duly following the rules in vogue to exchange seed materials.

## Abbreviations

*cp*: Chloroplast
tRNA: Transfer RNA
rRNA: Ribosomal RNA
SSR: Simple sequence repeats
SSC: Single copy region
LSC: Large single copy region
NAD-ME: NAD-dependent malic enzyme
NADP-ME: NADP-dependent malic enzyme
PEPCK: PEP carboxykinase
EMS: Ethyl methane sulfonate
SPAD: Soil plant analysis development
PPS: Portable photosynthesis system
CDS: Coding DNA sequence/ protein coding sequence
RSCU: Relative synonymous codon usage
LCB: Locally collinear block
IR: Inverted repeat regions
*cp*SSR: Chloroplast simple sequence repeats
