# Supplementary material for: Comparative chloroplast genomics of wild-type Panicum miliaceum cv. ATL1 and its M4 mutant line: insights for molecular breeding applications
Source: BMC Plant Biol. 2025 Aug 4;25:1023. doi: 10.1186/s12870-025-06999-5 (PMC12320348; doi:10.1186/s12870-025-06999-5)
Supplement: Supplementary file 1 — Supplementary Material 1.. [file 12870_2025_6999_MOESM1_ESM.docx]

**Supplementary Tables**

**Supplementary Table 1.** Details of annotated genes of cp genome in this study

| Name | Full Name | Name | Full Name | Name | Full Name |
| --- | --- | --- | --- | --- | --- |
| *psbA* | photosystem II protein D1 | *ndhJ* | NADH-plastoquinone oxidoreductase subunit J | *psbT* | photosystem II protein T |
| *trnK-UUU* | tRNA-Lys | *ndhK* | NADH-plastoquinone oxidoreductase subunit K | *psbN* | photosystem II protein N |
| *matK* | maturase K | *ndhC* | NADH-plastoquinone oxidoreductase subunit 3 | *psbH* | photosystem II phosphoprotein |
| *rps16* | ribosomal protein S16 | *trnV-UAC* | tRNA-Val | *petB* | cytochrome b6 |
| *psbK* | photosystem II protein K | *atpE* | ATP synthase CF1 epsilon subunit | *petD* | cytochrome b6/f complex subunit IV |
| *psbI* | photosystem II protein I | *atpB* | ATP synthase CF1 beta subunit | *rpoA* | RNA polymerase alpha subunit |
| *atpA* | ATP synthase CF1 alpha subunit | *rbcL* | ribulose 1,5-bisphosphate carboxylase/oxygenase large subunit | *rps11* | ribosomal protein S11 |
| *atpF* | ATP synthase CF0 subunit I | *psaI* | photosystem I subunit VIII | *rpl36* | ribosomal protein L36 |
| *atpH* | ATP synthase CF0 subunit III | *ycf4* | hypothetical protein | *rps8* | ribosomal protein S8 |
| *atpI* | ATP synthase CF0 subunit IV | *cemA* | chloroplast envelope membrane protein | *rpl14* | ribosomal protein L14 |
| *rps2* | ribosomal protein S2 | *petA* | cytochrome f | *rpl16* | ribosomal protein L16 |
| *rpoC2* | RNA polymerase beta' subunit | *psbJ* | photosystem II protein J | *rps3* | ribosomal protein S3 |
| *rpoC1* | RNA polymerase beta | *psbL* | photosystem II protein L | *rpl22* | ribosomal protein L22 |
| *rpoB* | RNA polymerase beta subunit | *psbF* | photosystem II cytochrome b559 beta subunit | *rps19* | ribosomal protein S19 |
| *petN* | cytochrome b6/f complex subunit VIII | *psbE* | photosystem II cytochrome b559 alpha subunit | *rpl2* | ribosomal protein L2 |
| *psbM* | photosystem II protein M | *petL* | cytochrome b6/f complex subunit VI | *rpl23* | ribosomal protein L23 |
| *psbD* | photosystem II protein D2 | *petG* | cytochrome b6/f complex subunit V | *infA* | Translational initiation factor |
| *psbC* | photosystem II CP43 chlorophyll apoprotein | *psaJ* | photosystem I subunit IX | *ndhB* | NADH-plastoquinone oxidoreductase subunit 2 |
| *rps14* | ribosomal protein S14 | *rpl33* | ribosomal protein L33 | *rps7* | ribosomal protein S7 |
| *psaB* | photosystem I P700 apoprotein A2 | *rps18* | ribosomal protein S18 | *rps12* | ribosomal protein S12 |
| *psaA* | photosystem I P700 apoprotein A1 | *rpl20* | ribosomal protein L20 | *trnI-GAU* | tRNA-Ile |
| *ycf3* | hypothetical chloroplast RF34 | *rps12* | ribosomal protein S12 | *trnA-UGC* | tRNA-Ala |
| *rps4* | ribosomal protein S4 | *clpP* | ATP-dependent Clp protease proteolytic subunit | *rplc* | Large ribosomal subunit protein |
| *trnL-UAA* | tRNA-Leu | *psbB* | photosystem II CP47 chlorophyll apoprotein | *ndhF* | NADH-plastoquinone oxidoreductase subunit 5 |
| *rpl32* | ribosomal protein L32 | *ndhI* | NADH-plastoquinone oxidoreductase subunit I | *rpsc* | RNA polymerase sigma factor SigC |
| *ccsA* | cytochrome c heme attachment protein | *ndhA* | NADH-plastoquinone oxidoreductase subunit 1 | *rps7* | ribosomal protein S7 |
| *ndhD* | NADH-plastoquinone oxidoreductase subunit 4 | *ndhH* | NADH-plastoquinone oxidoreductase subunit 7 | *ndhB* | NADH-plastoquinone oxidoreductase subunit 2 |
| *psaC* | photosystem I subunit VII | *rps15* | ribosomal protein S15 | *rpsd* | Small ribosomal subunit protein |
| *ndhE* | NADH-plastoquinone oxidoreductase subunit 4 L | *ndhc* | NAD(P)H-quinone oxidoreductase subunit 3 | *rpl23* | ribosomal protein L23 |
| *ndhG* | NADH-plastoquinone oxidoreductase subunit 6 | *trnA-UGC* | tRNA-Ala | *ycf15* | Putative uncharacterized protein |
| *ycf4* | PS I assembly protein | *ycf68c* | Uncharacterized protein | *orf42* |  |
| *psbZ* | PS II reaction protein Z | *rps15c* | Small ribosomal subunit protein uS19y | *rps7c* | Small ribosomal subunit protein eS7x |
| *rpsc* | Small ribosomal subunit protein | *ycf15c* | ATP synthase epsilon subunit C-terminal domain-containing protein | *rpl23c* | 60S ribosomal protein L23C |
| *rpl2b* | Large ribosomal subunit protein | *rps12b* | Small ribosomal subunit protein eS12B | *rps16b* | Small ribosomal subunit protein uS9 |
| *orf42* | Pvs-trna-like protein |  |  |  |  |

**Supplementary Table 2.** Details of tRNAs and rRNAs identified across the cp genomes investigated in this study

| **S. No.** | **tRNA** | **Reference PM** | **ATL1** | **TN*Pm*PEM001** |
| --- | --- | --- | --- | --- |
| 1 | *trnC-GCA* | + | + | + |
| 2 | *trnD-GUC* | + | + | + |
| 3 | *trnE-UUC* | + | + | + |
| 4 | *trnF-GAA* | + | + | + |
| 5 | *trnfM-CAUc* | + | + | + |
| 6 | *trnG-UCC* | + | + | + |
| 7 | *trnH-GUGc* | + | + | + |
| 8 | *trnI-CAUc* | + | + | + |
| 9 | *trnI-GAUc* | + | + | + |
| 10 | *trnK-UUU* | + | + | + |
| 11 | *trnL-CAAc* | + | + | + |
| 12 | *trnL-UAA* | + | + | + |
| 13 | *trnL-UAG* | + | + | + |
| 14 | *trnM-CAUc* | + | + | + |
| 15 | *trnN-GUUc* | + | + | + |
| 16 | *trnP-UGG* | + | + | + |
| 17 | *trnQ-UUG* | + | + | + |
| 18 | *trnR-ACGc* | + | + | + |
| 19 | *trnR-UCU* | + | + | + |
| 20 | *trnS-GCU* | + | + | + |
| 21 | *trnS-GGA* | + | + | + |
| 22 | *trnS-UGA* | + | + | + |
| 23 | *trnT-GGU* | + | + | + |
| 24 | *trnT-UGU* | + | + | + |
| 25 | *trnV-GACc* | + | + | + |
| 26 | *trnV-UAC* | + | + | + |
| 27 | *trnW-CCA* | + | + | + |
| 28 | *trnY-GUA* | + | + | + |
| 29 | *trnA-UGC* | - | + | + |
| 30 | *trnG-GCC* | - | + | + |
|  |  |  |  |  |

| **S. No.** | **rRNA** | **Reference PM** | **ATL1** | **TN*Pm*PEM001** |
| --- | --- | --- | --- | --- |
| 1 | *rrn23c* | + | + | + |
| 2 | *rrn16c* | + | + | + |
| 3 | *rrn5c* | + | + | + |
| 4 | *rrn4.5c* | + | + | + |

**Supplementary Table 3.** Details of SSRs investigated in this study with respect to motif frequency.

1. **Frequency of identified SSR motifs:**

**ATL1:**

| Repeats | 5 | 10 | 11 | 12 | 13 | 14 | 15 | Total |
| --- | --- | --- | --- | --- | --- | --- | --- | --- |
| A | - | 5 | 7 | 2 | 2 | - | - | 16 |
| C | - | 1 | - | - | - | - | - | 1 |
| G | - | - | 1 | - | - | - | - | 1 |
| T | - | 8 | 5 | 3 | - | 1 | 1 | 18 |
| AG | 1 | - | - | - | - | - | - | 1 |
| AT | 1 | - | - | - | - | - | - | 1 |
| TA | 4 | - | - | - | - | - | - | 4 |

**TN*Pm*PEM 001:**

| Repeats | 5 | 10 | 11 | 12 | 13 | 14 | 15 | Total |
| --- | --- | --- | --- | --- | --- | --- | --- | --- |
| A | - | 9 | 7 | 5 | 2 | - | - | 23 |
| C | - | 1 | - | - | - | - | - | 1 |
| G | - | - | 1 | - | - | - | - | 1 |
| T | - | 4 | 5 | - | - | - | 2 | 11 |
| AG | 1 | - | - | - | - | - | - | 1 |
| AT | 4 | - | - | - | - | - | - | 4 |
| TA | 1 | - | - | - | - | - | - | 1 |

1. **Frequency of classified repeat types:**

**ATL1:**

| Repeats | 5 | 10 | 11 | 12 | 13 | 14 | 15 | Total |
| --- | --- | --- | --- | --- | --- | --- | --- | --- |
| A/T | - | 13 | 12 | 5 | 2 | 1 | 1 | 34 |
| C/G | - | 1 | 1 | - | - | - | - | 2 |
| AG/CT | 1 | - | - | - | - | - | - | 1 |
| AT/AT | 5 | - | - | - | - | - | - | 5 |

**TN*Pm*PEM 001:**

| Repeats | 5 | 10 | 11 | 12 | 13 | 14 | 15 | Total |
| --- | --- | --- | --- | --- | --- | --- | --- | --- |
| A/T | - | 13 | 12 | 5 | 2 | - | 2 | 34 |
| C/G | - | 1 | 1 | - | - | - | - | 2 |
| AG/CT | 1 | - | - | - | - | - | - | 1 |
| AT/AT | 5 | - | - | - | - | - | - | 5 |

| Supplementary Table 4. Details of common SSR motifs found in ATL 1 and TN*Pm*PEM 001, but at different loci | | | | | | | | | | |
| --- | --- | --- | --- | --- | --- | --- | --- | --- | --- | --- |
| SSR Code | **Position (bp)(Start-End)** | | **Repeat motif** | | **Repeat size** | | **Melting temperature** | | **Forward primer** | **Reverse primer** |
|  | **ATL1** | **TN*Pm*PEM 001** | **ATL1** | **TN*Pm*PEM 001** | **ATL1** | **TN*Pm*PEM 001** | **Forward** | **Reverse** |  |  |
| Pm-CpSSR 024 | 134185-134463 | 29636-29914 | TA | TA | 10 | 10 | 60.179 | 60.107 | CGCGAGTTGCAGAGATGAGA | AACGTCCAGTGCCAAAGTCA |
| Pm-CpSSR 025 | 27011-27376 | 62299-62664 | AT | AT | 10 | 10 | 60.27 | 60.032 | GCGGATGCTGGCTATCTTACA | TCGACCAGATCCCCATGAGT |
| Pm-CpSSR 026 | 87200-87478 | 122488-122766 | TA | TA | 10 | 10 | 60.107 | 60.179 | AACGTCCAGTGCCAAAGTCA | CGCGAGTTGCAGAGATGAGA |
| Pm-CpSSR 027 | 1260-1510 | 36548-36798 | A | A | 11 | 11 | 60.319 | 59.654 | CCAATGTCAACCAAACCGGC | ACATAGGGAAAGTCGTGTGCA |
| Pm-CpSSR 028 | 3571-3887 | 38859-39175 | A | A | 10 | 10 | 59.745 | 58.854 | TAACCTTTCCCCGCATCAGG | ACTCGGCTTTTAAGTGCGAC |
| Pm-CpSSR 029 | 6037-6288 | 41324-41575 | T | T | 10 | 10 | 58.509 | 57.601 | TCTCACACTTCTTCGACTCGA | TGGTTAAGCAAAAACGATCTCG |
| Pm-CpSSR 030 | 7067-7344 | 42354-42631 | A | A | 11 | 11 | 60.036 | 59.675 | TCGCCAAATTACCCGAAGCT | GGGGATAGAAAGGCGGATCC |
| Pm-CpSSR 031 | 10271-10611 | 45558-45898 | G | G | 11 | 11 | 59.901 | 60.106 | GTGGCATTTATCACGCGCTT | CCCAACCATACATGTCCCCC |
| Pm-CpSSR 032 | 12392-12642 | 47679-47929 | A | A | 11 | 11 | 59.79 | 59.469 | TTGCCAAGGAGAAGATACGGG | TTGTCTCGGTGAATCCTCGG |
| Pm-CpSSR 033 | 14057-14333 | 49345-49621 | A | A | 10 | 10 | 58.79 | 60.092 | GGGCAGTACAGTCCCCATAA | GGAGTCCTCATCAGAGTCCTCT |
| Pm-CpSSR 034 | 19973-20223 | 55261-55511 | C | C | 10 | 10 | 60.272 | 58.866 | AGTGGATTTCCAGTCACAGGC | GAAAGCACGATTCTCCATAGGG |
| Pm-CpSSR 035 | 30751-31014 | 66039-66302 | A | A | 10 | 10 | 60.036 | 60.108 | AAAAGCTGCCCTACGAGGTC | CGGTAAACGGGGCTTCTGAT |
| Pm-CpSSR 036 | 33707-33994 | 68995-69282 | T | T | 11 | 11 | 57.914 | 59.964 | CGAAGTAATTCGGACGATTTCG | ACCTTCTGCTTCTGGCTGTC |
| Pm-CpSSR 037 | 38258-58536 | 73546-73824 | A | A | 12 | 12 | 60.035 | 60.11 | TGCCATAATGTGCCGTTCCT | CGTGGCTCTTTCCATTGTGC |
| Pm-CpSSR 038 | 44296-44554 | 79584-79842 | T | T | 10 | 10 | 59.657 | 58.759 | TCGTGAAGTGTCCTTCCTTCC | CACACAAGCAATGGAGAGCA |
| Pm-CpSSR 039 | 45841-46157 | 81129-81445 | A | A | 13 | 13 | 59.796 | 59.903 | TGGCGTAACGTACCTTATGCA | CAATGCTACGCCTTGAACCG |
| Pm-CpSSR 040 | 47805-48112 | 83093-83400 | T | T | 11 | 11 | 57.028 | 59.962 | TTTGATATGGCTCGGACGAA | TTTTTCTCTCCCCCGCCTTC |
| Pm-CpSSR 041 | 50996-51296 | 86284-86584 | T | T | 15 | 15 | 60.09 | 60.25 | TGGTACCATATAGAAGGGGCCA | TGGGCAATGAGTTTCGACGT |
| Pm-CpSSR 042 | 52345-52598 | 87633-87886 | A | A | 13 | 13 | 60.874 | 58.903 | TTCTATGGCGCAATCGACCG | TGATGAAAGAGCCCAATGCAG |
| Pm-CpSSR 043 | 56071-56324 | 91359-91612 | T | T | 11 | 11 | 57.626 | 59.962 | ACACACGGTGTACGCATTAT | TTCGTACTCCGGGGTGTAGT |
| Pm-CpSSR 044 | 45240-45505 | 80528-80793 | (A)_11_g(A)_10_ | (A)_11_g(A)_10_ | 22 | 22 | 59.821 | 58.888 | TGAGGCCACATCAATCGAGG | GACCTCCTCGATTGTAGCCA |

**Supplementary Table 5:** Codon preference analysis with the three investigated samples

| ATL1 | | | |  | TN*Pm*PEM 001 | | | |
| --- | --- | --- | --- | --- | --- | --- | --- | --- |
| Codon | **Amino acid** | **Count** | **RSCU** |  | **Codon** | **Amino acid** | **Count** | **RSCU** |
| TTT | F | 1972 | 1.212 |  | **TTT** | F | 1736 | 1.176 |
| TTC | F | 1282 | 0.788 |  | **TTC** | F | 1216 | 0.824 |
| TTA | L | 1012 | 1.283 |  | **TTA** | L | 929 | 1.220 |
| TTG | L | 826 | 1.047 |  | **TTG** | L | 962 | 1.263 |
| CTT | L | 1051 | 1.332 |  | **CTT** | L | 926 | 1.216 |
| CTC | L | 618 | 0.783 |  | **CTC** | L | 569 | 0.747 |
| CTA | L | 787 | 0.998 |  | **CTA** | L | 756 | 0.993 |
| CTG | L | 439 | 0.557 |  | **CTG** | L | 427 | 0.561 |
| ATT | I | 1539 | 1.283 |  | **ATT** | I | 1427 | 1.187 |
| ATC | I | 837 | 0.728 |  | **ATC** | I | 918 | 0.764 |
| ATA | I | 1187 | 0.989 |  | **ATA** | I | 1261 | 1.049 |
| ATG | M | 793 | 1.000 |  | **ATG** | M | 795 | 1.000 |
| GTT | V | 770 | 1.413 |  | **GTT** | V | 618 | 1.279 |
| GTC | V | 382 | 0.701 |  | **GTC** | V | 371 | 0.768 |
| GTA | V | 619 | 1.136 |  | **GTA** | V | 623 | 1.289 |
| GTG | V | 409 | 0.750 |  | **GTG** | V | 321 | 0.664 |
| TCT | S | 1044 | 1.486 |  | **TCT** | S | 938 | 1.358 |
| TCC | S | 770 | 1.096 |  | **TCC** | S | 793 | 1.148 |
| TCA | S | 711 | 1.012 |  | **TCA** | S | 732 | 1.060 |
| TCG | S | 492 | 0.700 |  | **TCG** | S | 546 | 0.791 |
| CCT | P | 634 | 1.174 |  | **CCT** | P | 661 | 1.150 |
| CCC | P | 548 | 1.014 |  | **CCC** | P | 541 | 0.941 |
| CCA | P | 674 | 1.248 |  | **CCA** | P | 728 | 1.266 |
| CCG | P | 305 | 0.565 |  | **CCG** | P | 370 | 0.643 |
| ACT | T | 680 | 1.282 |  | **ACT** | T | 630 | 1.210 |
| ACC | T | 544 | 1.025 |  | **ACC** | T | 544 | 1.045 |
| ACA | T | 523 | 0.986 |  | **ACA** | T | 573 | 1.101 |
| ACG | T | 375 | 0.707 |  | **ACG** | T | 335 | 0.644 |
| GCT | A | 525 | 1.332 |  | **GCT** | A | 490 | 1.241 |
| GCC | A | 338 | 0.858 |  | **GCC** | A | 376 | 0.953 |
| GCA | A | 452 | 1.147 |  | **GCA** | A | 447 | 1.132 |
| GCG | A | 261 | 0.662 |  | **GCG** | A | 266 | 0.674 |
| TAT | Y | 1227 | 1.321 |  | **TAT** | Y | 1147 | 1.280 |
| TAC | Y | 630 | 0.679 |  | **TAC** | Y | 645 | 0.720 |
| TAA | * | 942 | 1.185 |  | **TAA** | * | 1061 | 1.212 |
| TAG | * | 762 | 0.958 |  | **TAG** | * | 812 | 0.928 |
| CAT | H | 759 | 1.404 |  | **CAT** | H | 728 | 1.291 |
| CAC | H | 322 | 0.596 |  | **CAC** | H | 400 | 0.709 |
| CAA | Q | 924 | 1.358 |  | **CAA** | Q | 858 | 1.332 |
| CAG | Q | 437 | 0.642 |  | **CAG** | Q | 430 | 0.668 |
| AAT | N | 1532 | 1.411 |  | **AAT** | N | 1498 | 1.329 |
| AAC | N | 639 | 0.589 |  | **AAC** | N | 757 | 0.671 |
| AAA | K | 1825 | 1.305 |  | **AAA** | K | 2163 | 1.336 |
| AAG | K | 971 | 0.695 |  | **AAG** | K | 1076 | 0.664 |
| GAT | D | 901 | 1.401 |  | **GAT** | D | 855 | 1.381 |
| GAC | D | 385 | 0.599 |  | **GAC** | D | 383 | 0.619 |
| GAA | E | 1248 | 1.369 |  | **GAA** | E | 1325 | 1.338 |
| GAG | E | 575 | 0.631 |  | **GAG** | E | 655 | 0.662 |
| TGT | C | 558 | 1.118 |  | **TGT** | C | 553 | 1.096 |
| TGC | C | 440 | 0.882 |  | **TGC** | C | 456 | 0.904 |
| TGA | * | 681 | 0.857 |  | **TGA** | * | 753 | 0.860 |
| TGG | W | 692 | 1.000 |  | **TGG** | W | 699 | 1.000 |
| CGT | R | 361 | 0.671 |  | **CGT** | R | 323 | 0.608 |
| CGC | R | 255 | 0.474 |  | **CGC** | R | 235 | 0.442 |
| CGA | R | 494 | 0.918 |  | **CGA** | R | 541 | 1.018 |
| CGG | R | 362 | 0.673 |  | **CGG** | R | 330 | 0.621 |
| AGT | S | 669 | 0.952 |  | **AGT** | S | 623 | 0.902 |
| AGC | S | 530 | 0.754 |  | **AGC** | S | 511 | 0.740 |
| AGA | R | 1057 | 1.964 |  | **AGA** | R | 1135 | 2.135 |
| AGG | R | 700 | 1.301 |  | **AGG** | R | 626 | 1.177 |
| GGT | G | 522 | 0.908 |  | **GGT** | G | 482 | 0.873 |
| GGC | G | 347 | 0.604 |  | **GGC** | G | 351 | 0.636 |
| GGA | G | 849 | 1.477 |  | **GGA** | G | 827 | 1.498 |
| GGG | G | 581 | 1.011 |  | **GGG** | G | 549 | 0.994 |

_RSCU: relative synonymous codon usage_
